# A Mysterious Gram-Positive Rods

**DOI:** 10.1155/2012/841834

**Published:** 2012-08-22

**Authors:** Javzandulam Natsag, Zaw Min, Yasir Hamad, Bassel Alkhalil, Atiq Rahman, Richard Williams

**Affiliations:** Department of Internal Medicine, MedStar Harbor Hospital, 3001 South Hanover Street, Baltimore, MD 21225, USA

## Abstract

We encountered a patient with a history of intravenous drug use presenting with fever, malaise and nausea who was found to have cavitary lung lesions. Unexpectedly, gram positive rods grew out on day five on multiple blood cultures, which were later identified as *Mycobacterium fortuitum*. The patient underwent transesophageal echocardiogram, which showed aortic and tricuspid valve vegetations. Liver biopsy demonstrated granulomatous hepatitis. Interestingly, serum alkaline phosphatase level fell with antibiotic treatment. *Mycobacterium fortuitum* is ubiquitous worldwide, being found in tap water, and soil. *M. fortuitum* is usually considered as a contaminant. Disseminated infection caused by this bacterium in an immunocompetent host is extremely rare. Most of the disseminated infections have been reported in immune-deficient patients. In immunocompetent people, *M. fortuitum* causes human infection primarily by direct inoculation, including localized post-traumatic and surgical wound infections, and catheter-related sepsis. Our patient, an HIV-negative intravenous drug user, had *Mycobacterium fortuitum* sepsis associated with infective endocarditis, septic pulmonary emboli, and granulomatous hepatitis. Interestingly, the patient admitted using tap water occasionally for mixing heroin when her sterile water ran out, which we thought was the likely source of *M. fortuitum*.

## 1. Introduction

We encountered a patient with a history of intravenous drug use (IDU) presenting with fever, malaise, and nausea who was found to have cavitary lung lesions. Unexpectedly, gram positive rods (GPR) grew out on day five blood cultures, leading us on a whirlwind adventure to the right diagnosis and treatment.

## 2. Case

A 49-year-old female with active intravenous heroin use was admitted with fever, chills, night sweats, malaise, nausea, and 15 lb weight loss for 2 months. About a month prior to admission, the patient had a foul smelling, draining skin abscess in the left arm at the site of heroin injection, which was treated with a course of trimethoprim/sulfamethoxazole prescribed in the emergency room. Although the abscess resolved, her constitutional symptoms worsened. She reported no cough, vomiting, diarrhea, abdominal pain, or dysuria and was taking no regular medications. She used heroin daily and had a twenty pack-year smoking history. She had been incarcerated for 9 months about five years prior to admission.

On physical examination, the patient was cachectic and malnourished. She was awake, alert, fully oriented and in no acute distress. Temperature was 39.4°C, blood pressure 115/69, heart rate 127, respiratory rate 20, and oxygen saturation 99% on ambient air. The area of the previous injection site infection was healed. Multiple needle track marks in the cubital fossae were noted. There was no cervical and axillary lymphadenopathy, and the lungs were clear to auscultation. Cardiovascular exam showed a regular tachycardia with normal S1 and S2. There were no murmurs or extra heart sounds. Abdominal exam was significant for mild right-upper-quadrant tenderness to deep palpation and a liver span of 14 cm in the right midclavicular line.

**Figure 1:**
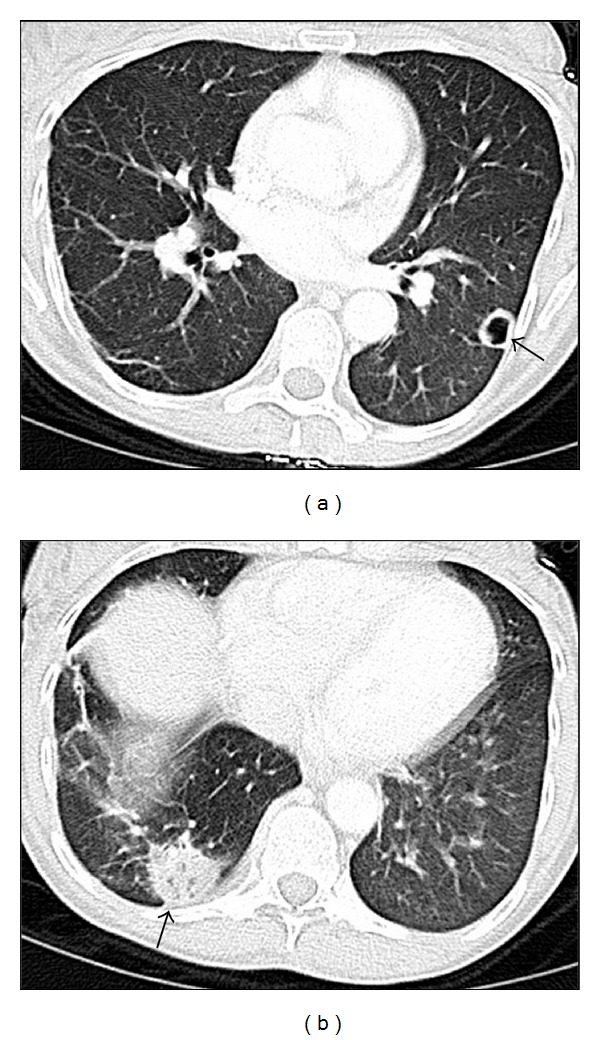
Chest CT shows a peripheral 1.7 cm cavitary nodule in the left lower lobe (a), and a peripheral 3 cm mass-like infiltrate in the right lower lobe (b).

Laboratory showed a leukocytosis of 11.2, a markedly elevated serum alkaline phosphatase of 1210 unit/L (normal 38–126) and gamma-glutamyl transpeptidase (GGT) 957 unit/L (normal 12–43), as well as slightly elevated AST 125 unit/L, ALT 55 unit/L, and total bilirubin 1.5 mg/dL with direct bilirubin 0.9 mg/dl. Chest X-ray showed a nodule in the left lower lung. Intravenous contrast-enhanced chest CT demonstrated a peripheral 1.7 cm cavitary nodule in the left lower lobe and a peripheral 3 cm mass-like infiltrate in the right lower lobe (Figure 1). Abdominal ultrasound demonstrated hepatomegaly without focal abnormality. The patient was empirically started on vancomycin for suspected staphylococcal endocarditis and was placed on respiratory isolation for possible tuberculosis. Since the patient did not produce sputum, bronchoscopy was performed, and an AFB smear as well as bronchoalveolar lavage culture were negative. Transthoracic echocardiogram showed normal systolic function without vegetation. Hepatitis C serology was positive, and HIV was negative. The patient continued to be febrile and tachycardic on day 3, her blood pressure fell to 85/44, and piperacillin/tazobactam was added to vancomycin, along with intravenous fluid resuscitation. Although blood culture was negative initially, gram positive rods, which appeared to be branching, were grown in multiple cultures after 5 days. Nocardia was suspected, therefore, trimethoprim/sulfamethoxazole (TMP/SMX) was started, and vancomycin and piperacillin/tazobactam were continued. Subsequently, the infectious disease consult recommended switching from vancomycin to linezolid to cover adequately for possible disseminated Nocardia infection. Therefore, the patient was placed on linezolid, piperacillin/tazobactam, and TMP/SMX. After the antibiotics regimen was initiated, the patient became afebrile and normotensive and felt much better. On day 14, the patient was discharged home with oral linezolid and TMP/SMX; however, she failed to keep her follow-up appointment and did not take her medications. She was readmitted 14 days later with the recurrence of all prior symptoms.

**Figure 2:**
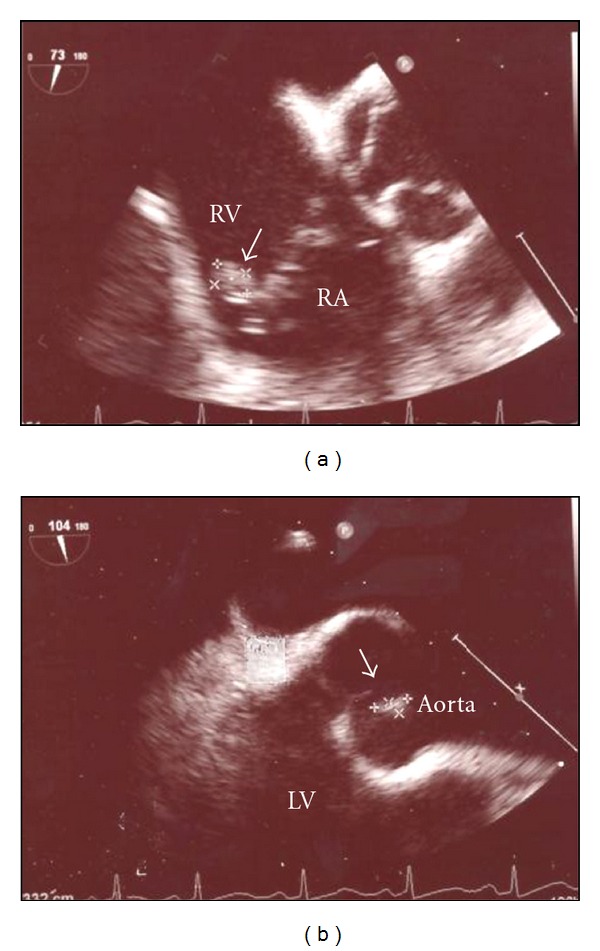
Transesophageal echocardiogram showing tricuspid (a) and aortic valve vegetations (b) consistent with endocarditis.

**Figure 3:**
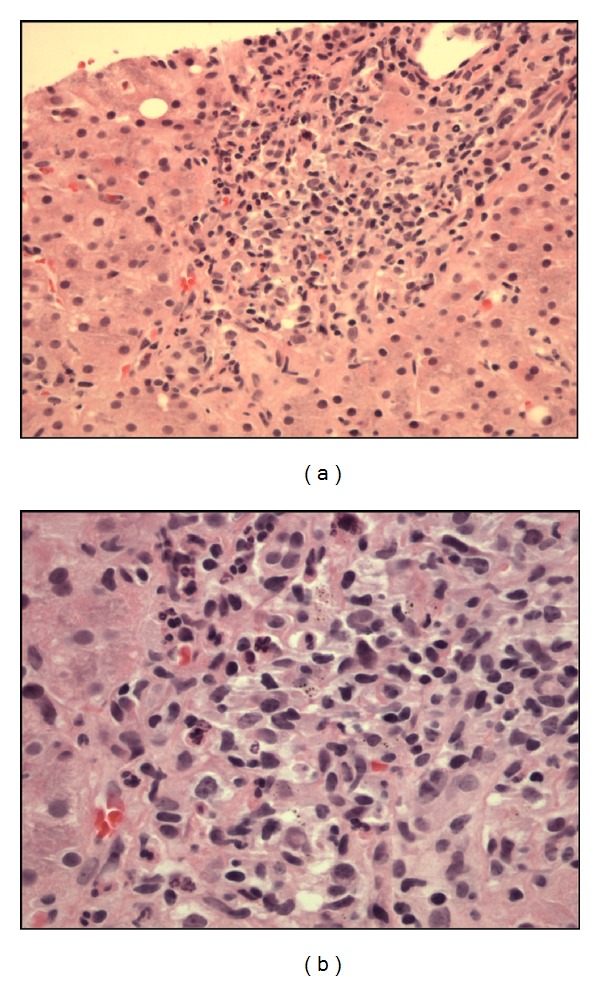
(a) Liver biopsy specimen on H&E stain showing noncaseating granuloma. (b) On greater magnification, the granuloma consists of mixture of inflammatory cells, including epithelioid histiocytes and lymphocytes.

**Figure 4:**
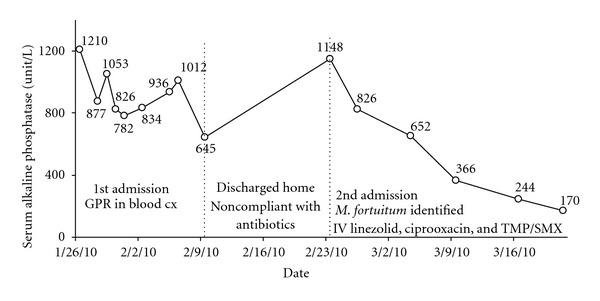
Serum alkaline phosphatase level trend in response to antibiotic treatment. (Normal serum level is 38–126 unit/L.)

Approximately 15 days after the first admission, the reference lab reported that the gram positive rods were also acid fast and identified them as *Mycobacterium fortuitum*. The organism was grown in 6 aerobic bottles of a total of 15 blood cultures obtained during the two hospital admissions. It was sensitive to linezolid, imipenem, ciprofloxacin, amikacin, doxycycline, and sulfamethoxazole; and was resistant to clarithromycin, cefoxitin, and tobramycin. During the second admission, the patient was placed on intravenous linezolid and ciprofloxacin, and oral TMP/SMX with clinical improvement. She underwent transesophageal echocardiogram, which showed aortic and tricuspid valve vegetations (Figure 2). A liver biopsy showed granulomatous hepatitis (Figure 3). No organisms could be identified on the liver biopsy culture. However, serum alkaline phosphatase level fell with antibiotic treatment (Figure 4).

The patient completed a six-week course of intravenous antibiotics and was discharged on oral bactrim and ciprofloxacin with goal of continuing the antibiotics for 6–12 months. She was counseled regarding options for treating her narcotic addiction.

## 3. Discussion

*Mycobacterium fortuitum* is ubiquitous worldwide, being found in tap water, soil, and dust [1]. It belongs to the rapidly-growing nontuberculous mycobacteria group, growing in culture within a week, rapidly as compared to other mycobacteria [2]. *M. fortuitum* is usually considered as a contaminant [1]. Disseminated infection caused by this bacterium in an immunocompetent host is extremely rare. Most of the disseminated infections have been reported in immune-deficient patients [3]. In immunocompetent people, *M. fortuitum* causes human infection primarily by direct inoculation, including localized posttraumatic and surgical wound infections, and catheter-related sepsis [1–3]. Rarely, other infections occur such as keratitis, prosthetic valve endocarditis, cervical lymphadenitis, and pulmonary disease [2, 3]. Our patient, an HIV-negative intravenous drug user, had *Mycobacterium fortuitum* sepsis associated with infective endocarditis, septic pulmonary emboli, and granulomatous hepatitis.

Our patient most likely became infected with *M. fortuitum* through intravenous drug use, with bacteremia and endocarditis. Upon further questioning, the patient was found to be enrolled in the needle exchange program, which provides sterile needles, water, and bleach for IDU in the city of Baltimore. Our patient reported having been meticulous in following aseptic rules. For instance, when she ran out of sterile needles, she cleaned her used needles with bleach. However, she occasionally used tap water for mixing heroin when sterile water ran out. Interestingly, tap water is one of the common sources of *M. fortuitum*. Moreover, mycobacterium is resistant to low concentration of bleach. Therefore, our patient likely was selectively injecting *M. fortuitum*, rather than the more common *Staphylococcus aureus*, which would have been killed by the bleach and not generally present in tap water.

Endocarditis caused by *M. fortuitum* is rare [4], with only 20 cases having been reported to date [5]. In the majority of these cases, the infection occurred on prosthetic valves, both mechanical and biologic [5]. Native valve endocarditis caused by this organism is even rarer, with only 4 cases reported to date [5–8] (Table 1). Our case is the fourth case report of *M. fortuitum* granulomatous hepatitis [9–11] and the second case report of *M. fortuitum* double native valve endocarditis [5]. Moreover it is the first case report of *M. fortuitum* native valve endocarditis and granulomatous hepatitis—presenting in the same patient.

*M. fortuitum* is a thin, branching, and gram positive bacillus. Nocardia is typically the first organism suspected when branching gram positive bacilli are identified, which can lead to a delay in proper therapy [12]. Ziehl-Nielsen acid-fast stain can be helpful to differentiate these organisms. However, rapidly growing mycobacteria are more easily decolorized by acid alcohol, a property that makes them more difficult to stain than the slowly growing mycobacteria [12, 13]. As result, acid-fast stain can be variable.

Empiric treatment for severe disseminated *M. fortuitum* includes parenteral amikacin plus two of the following drugs: tobramycin, cefoxitin, and levofloxacin. An alternative is that a combination of all three of the latter drugs may be used. Susceptibility testing is very important in choosing antibiotics because of differences in susceptibilities among species of rapidly growing mycobacterium. For severe disease, at least two agents to which the *M. fortuitum* isolate is susceptible should be initiated and continued for 2–6 weeks until clinical improvement is evident, followed by oral therapy with two effective agents for 6–12 months [2].

## 4. Conclusion

Mycobacterium species should be included in the differential diagnosis of GPR bacteremia in patients with history of intravenous drug use, as well as catheter, and dialysis-related sepsis. AFB stain can be helpful if branching GPRs are identified in the aerobic bottles of the blood smear. Granulomatous hepatitis, which could develop secondary to mycobacterial bacteremia, presents with disproportionate elevation of the serum alkaline phosphatase.

## Figures and Tables

**Table 1:** Summary of previously published cases of *M. fortuitum* native endocarditis.

Authors	Age	Valves affected	HIV Status	Previous procedure	Outcome
Singh et al. [7]	54	Aortic	Unknown	Hemodialysis	Death
Spell et al. [6]	47	Aortic	Positive	IDU	Death
Kuruvila et al. [8]	20	Mitral	Unknown	Balloon mitral valvotomy	Death
Collison and Trehan [5]	50	Mitral Aortic	Unknown	Percutaneous coronary intervention and stent placement	Alive
Our case	49	Aortic Tricuspid	Negative	IDU	Alive

## References

[1] Brown-Elliott BA, Wallace RJ (2002). Clinical and taxonomic status of pathogenic nonpigmented or late-pigmenting rapidly growing mycobacteria. *Clinical Microbiology Reviews*.

[2] Griffith DE, Wallace RJ (2009). *Rapidly Growing Mycobacterial Infections in HIV-Negative Patients: UpToDate*.

[3] Wallace RJ, Swenson JM, Silcox VA, Good RC, Tschen JA, Stone MS (1983). Spectrum of disease due to rapidly growing mycobacteria. *Reviews of Infectious Diseases*.

[4] Olalla J, Pombo M, Aguado JM (2002). *Mycobacterium fortuitum* complex endocarditis—case report and literature review. *Clinical Microbiology and Infection*.

[5] Collison SP, Trehan N (2006). Native double-valve endocarditis by *Mycobacterium fortuitum* following percutaneous coronary intervention. *Journal of Heart Valve Disease*.

[6] Spell DW, Szurgot JG, Greer RW, Brown JW (2000). Native valve endocarditis due to *Mycobacterium fortuitum* biovar fortuitum: case report and review. *Clinical Infectious Diseases*.

[7] Singh M, Bofinger A, Cave G, Boyle P (1992). *Mycobacterium fortuitum* endocarditis in a patient with chronic renal failure on hemodialysis. *Pathology*.

[8] Kuruvila MT, Mathews P, Jesudason M, Ganesh A (1999). *Mycobacterium fortuitum* Endocarditis and Meningitis after Balloon Mitral Valvotomy. *Journal of Association of Physicians of India*.

[9] Brannan DP, DuBois RE, Ramirez MJ, Ravry MJ, Harrison EO (1984). Cefoxitin therapy for *Mycobacterium fortuitum* bacteremia with associated granulomatous hepatitis. *Southern Medical Journal*.

[10] Montoliu J, Gatell JM, Bonal J (1985). Disseminated visceral infection with *Mycobacterium fortuitum* in a hemodialysis patient. *American Journal of Nephrology*.

[11] Ingram CW, Tanner DC, Durack DT, Kernodle GW, Corey GR (1993). Disseminated infection with rapidly growing mycobacteria. *Clinical Infectious Diseases*.

[12] Smith MB, Schnadig VJ, Boyars MC, Woods GL (2001). Clinical and pathologic features of *Mycobacterium fortuitum* infections: an emerging pathogen in patients with AIDS. *American Journal of Clinical Pathology*.

[13] McFarland EJ, Kuritzkes DR (1993). Clinical features and treatment of infection due to *Mycobacterium fortuitum*/chelonae complex. *Current Clinical Topics in Infectious Diseases*.

